# An adult learning perspective on disability and microfinance: The case of Katureebe

**DOI:** 10.4102/ajod.v5i1.215

**Published:** 2016-08-24

**Authors:** Ephraim L. Nuwagaba, Peter N. Rule

**Affiliations:** 1 Department of Adult and Community Education, Kyambogo University, Uganda; 2 School of Education, University of KwaZulu-Natal, South Africa

## Abstract

**Background:**

Despite Uganda’s progress in promoting affirmative action for persons with disabilities and its strategy of using microfinance to fight poverty, access to microfinance services by persons with disabilities is still problematic due to barriers, characterised by discrepancies between policies and practices. Regarding education, the affirmative action in favour of learners with disabilities has not translated into actual learning opportunities due to personal and environmental barriers.

**Objectives:**

The study on which this article is based investigated the non-formal and informal adult learning practices regarding microfinance that persons with disabilities engaged in. This article seeks to illuminate the barriers that a person with a visual impairment encountered while learning about and engaging with microfinance and the strategies that he developed to overcome them.

**Methods:**

This was a case study, framed within the social model of disability and critical research paradigm. Data were collected through in-depth interviews of a person with visual impairment and observations of the environment in which adult learning and engagement with Savings and Credit Cooperative Organisations (SACCOs) occurred.

**Results:**

Findings indicate that the person with a visual disability faced barriers to learning about microfinance services. He experienced barriers in an integrated manner and developed strategies to overcome these barriers. The barriers and strategies are theorised using the social model of disability.

**Conclusion:**

The case of a person with visual impairment suggests that persons with disabilities face multiple barriers regarding microfinance, including social, psychological and educational. However, his own agency and attitudes were also of importance as they influenced his learning. Viewing these barriers as blockades can lead to non-participation in learning and engagement with microfinance whereas viewing them as surmountable hurdles can potentially motivate participants to succeed in learning about and engaging with microfinance.

## Introduction

Access to microfinance is a vital but problematic issue for persons with disabilities who wish to pursue their own businesses. In Uganda, microfinance is a strategy that is increasingly being used in the fight against poverty. Unfortunately, persons with disabilities are faced with challenges while learning about and engaging with microfinance (Nuwagaba 2012). This is despite the existence of the Community-Based Rehabilitation (CBR) Guidelines of 2010 and the United Nations Convention on the Rights of people with disabilities (UNCRPD) (UN 2006) that advocate inclusiveness. Similarly, the good policies that exist in Uganda are not matched by good practices (Abimanya-Ochom & Mannan 2014).

Katureebe’s exemplary case has been selected because it provides rich experiences of his learning and insights into many of the barriers that people with visual impairments face while learning about microfinance and the strategies to address them. Understanding the case of a person with disability’s learning experiences and the challenges he faces while learning about microfinance can provide insight into the complexity of the interplay between disability and adult learning and provide a basis for improving education provision for people with visual disabilities. The case also provides a glimpse of the complex relationships between adult education, disability and microfinance.

The purpose of this article is to illuminate the barriers that a person with a visual impairment encountered while learning about microfinance and the strategies that he developed to overcome them. It provides an understanding of the interplay between unfavourable physical and psychological factors, learning environment and inadequate finances. We start with the research context and then frame the study within relevant literature and theory. This is followed by an explanation of the methodology employed, a profile of Katureebe and the presentation of results focusing on adult learning practices, the barriers encountered and strategies adopted. We discuss the findings in relation to the social model of disability. We conclude with some reflections on the implications regarding adults with disabilities, learning and microfinance.

### Background

#### Research context

The World Report on Disability (WHO 2011) estimates that 15.3% of the world population is disabled and is likely to have lower educational and employment opportunities. WHO (2010b) confirms that in Uganda, as in many developing countries, persons with disabilities have low literacy levels. While the net enrolment in primary education stands at 81.1% (UBOS 2013), only 15% of children with disabilities are able to access education, 5% through inclusive schools and 10% through special schools (UNICEF 2012, cited in Nyende 2012).

This limits persons with disabilities’ acquisition of what WHO (2010b:1) calls ‘foundation skills’, including those required to successfully run a business. Lwanga-Ntale (2003) confirms that in Uganda many persons with disabilities are of low formal education levels and hardly have any formal employment opportunities. For those who wish to start or improve their own businesses, accessing microfinance loans requires literacy skills such as filling in loan application forms, calculating interest on loans and keeping records. Persons with disabilities’ access to education may therefore enhance their access to microfinance services; conversely, lack of education may limit access. It is likely that the income situation of persons with disabilities would change if they had access to microfinance loans (MoFPED 2008) and appropriate adult learning opportunities (Nuwagaba *et al*. 2012; Association of Microfinance Institutions [AMFIU] 2010). The need to provide educational opportunities for such a large and deprived group cannot be ignored. Unfortunately, in Uganda, as in many African countries, persons with disabilities hardly benefit from adult education provision (Nuwagaba 2012).

Despite the challenges, some persons with disabilities, including those who are semi-literate, do access microfinance services (Nuwagaba *et al*. 2012). However, the way they acquire the knowledge and skills to improve their livelihood is largely unknown and undocumented. The microfinance services in this study were those provided by Savings and Credit Cooperative Organisations (SACCOs). According to the Executive Director, Microfinance Centre, ‘SACCOs are member-owned, member-managed and member-used; community based and much closer to the poor than mainstream MFIs [*our addition: micro finance institutions*]’ (Nuwagaba 2013:10).

Unfortunately, the field of adult education and disability in African contexts is under-researched (Belanger & Blais 1995; Omolewa 1995) and especially how disability ‘affects, impacts and/or constrains the adult learning context’ (Clark 2006:310). This is reflected in sub-Saharan countries’ recommendation 28 for the International Conference in Adult Education (CONFINTEA) VI that ‘all adult learning and education programmes should take into consideration the special needs of disabled learners’ (Aitchison & Alidou 2009:67).

#### Objectives

This article builds on Nuwagaba’s (2013) study by engaging in more in-depth analysis to bring out the complexities of the barriers experienced by a person with visual disabilities during learning processes and the strategies employed to overcome them.

### Literature review

#### Understanding the unique characteristics of adult learners with disabilities

While adult learners with disabilities engage holistically in adult learning processes, like learners without disabilities (Rogers 2003), they are a minority group whose voices are often silenced (Clark 2006). Their self-identity and how they are viewed by society play a much bigger negative role in their learning than for those without disabilities, because society often regards them as inadequate. Some of them have internalised their oppression and, as Freire (1972) argues, those who get to this stage believe their situation cannot be changed.

On the other hand, there is evidence that some adult learners with disabilities have a strong motivation to learn. Rule & Modipa (2012) investigated the attitudes of adult learners with disabilities regarding education in a non-governmental organisation setting in South Africa. They found that they had negative childhood experiences of education and suffered stigmatisation. As adults, however, they had a strong desire to learn and attempted to affirm their position in society as people with potential just like anyone else.

In addition to the social barriers that exclude them from microfinance services, adult learners with disabilities may also be hindered by lack of self-esteem and self-confidence, themselves internalised consequences of social barriers (WHO 2010a). Their families’ or own expectations of an entitlement to charity may reinforce this exclusion. Additionally, adult learners, especially those who have faced discrimination and inequality, have strong emotions which can either support or hinder learning (Zembylas 2008). In his study on distance learners, Zembylas describes attitudes that arise out of inequality and discrimination as ‘us versus them’. Adult learners with disabilities may fit this description as they face discrimination and marginalisation. For such learners, who may feel persecuted and defensive, learning with learners without disabilities can be problematic unless society takes explicit measures to accommodate them (Zembylas 2008).

Adult education literature recognises the uniqueness of adult learners with disabilities (Covington 2004; DuBois 1998; Gadbow 2002; Horton & Hall 1998; Polson & White 2000). Clark (2006) identifies such learners as individuals with unique or special needs that can be addressed through provision of sign language interpretation, note-taking services, assistive technology and separate and/or extra time for exams – services that can be provided by society. Although Clark’s conceptualisation relates to formal learning, it is, apart from examinations, relevant to non-formal adult learning. Relevant content and the use of facilitators, who are able to recognise the characteristics of adult learners with disabilities and employ appropriate methodologies, can also help to address their learning needs.

#### Framing the study in theory: The social model of disability

The social model of disability was developed as a reaction and alternative to the medical model, which defined disability in medical terms and located it as a problem of the ‘patient’. According to the social model, economic, cultural, attitudinal, physical and social barriers stop people with impairments from participating fully in society, and so create disabilities (Germon 2000; Ndeezi 2004; Oliver 1996; Truman 2000). Society is viewed as the problem, not the person with impairment. The social model fits in well with the agenda of the disability movement in Uganda which advocates for the removal of barriers to the participation of persons with disabilities in all spheres of life (Ndeezi 2004; Nuwagaba 2012). Uganda’s National Policy on Disability understands disability as ‘permanent and substantial functional limitation of daily life activities caused by physical, mental or sensory impairment and environmental barriers resulting in limited participation’ (MoGLSD 2006:28). This definition focuses on the disabling environment although the impairment is not ignored. However, the social model is criticised for downplaying the role of impairment and personal experiences (Mercer 2002; Scully 2008 cited in Rule & Modipa 2012).

The social model of disability resonates well with the critical research paradigm, which was adopted in this study, and which can be used to understand and contribute to reshaping oppressive structures and processes in society such as those experienced by persons with disabilities (Henning, Van Rensburg & Smit 2004). The social model is relevant to this study because it facilitated the understanding of Katureebe’s experiences and the influence of environmental factors. This understanding can be used to work towards making such environmental factors more favourable to persons with disabilities and improving their quality of life. The framework helps to contextualise the learning and engagement with SACCOs in this study by locating them as a relation between the individual person with impairment and what Schneider (2006) calls environmental factors, such as the influences of the conditions of the SACCOs, families and the communities in which they live, work and learn.

## Research method and design

The study adopted a qualitative case study approach (Rule & John 2011; Simons 2009; Yin 2003) within the critical research paradigm (Henning *et al*. 2004). This paradigm was adopted as an appropriate theoretical frame because the study investigated the marginalisation of a person with visual disabilities involved in non-formal and informal adult learning and the strategies he employed to transform this situation. The transformation focused on redressing discrimination, inequalities, barriers, and social structures and systems, which according to Oliver (1996), Truman (2000) and Ndeezi (2004) (cited in Nuwagaba & Rule’s 2015, p. 258), prevent people with impairments from full social participation. In this way, the critical research paradigm and the social model of disability complement each other.

A case study was adopted because, with its focus on the specific case, it can provide a rich description of a particular instance, examining the complex relations within the case and in relation to its context (Rule & John 2011), as well as yield insights into the relation between the case and what it is a case of (Simons 2009). Walter (2009:514) also affirms that case studies can be used to ‘provide insight into educational issues and processes’, in this instance, the adult learning practices of a person with visual disability. In addition, case study research can apply and develop theory (Rule & John 2015) such as, in this study, the social model of disability.

The sampling approach used in the study combined purposive and snowball sampling (Chilisa & Preece 2005; Niewenhuis 2007). Purposive sampling was used to identify the SACCOs serving the persons with disabilities in the western Ugandan district of Bushenyi because, according to UCA (2009), the district had vibrant SACCOs. As SACCOs do not easily release information about their clients, snowball sampling was used to identify Katureebe. A leader of persons with disabilities was contacted and she helped identify some persons with disabilities involved in SACCOs and these in turn identified others, including Katureebe. Katureebe was selected due to his long involvement in SACCOs and his role as a leader among persons with disabilities using microfinance. We do not view his experiences as representative of, or generalisable to, all persons with disabilities involved in microfinance, as we indicate in our ‘Discussion’ section. Nevertheless, this case provides valuable insights into the social model of disability in relation to disability, adult learning and microfinance.

Data collection involved the use of loosely structured interviews and observations. Katureebe was interviewed three times between 2012 and 2013, and each time the interview lasted about one and a half hours. The interview questions focused on type and degree of impairment and their effect on learning and engagement with SACCOs, the challenges faced during learning processes and the conduciveness of the learning venues. The interviews were conducted in the local language and tape-recorded. They were then transcribed and translated into English by the researcher and a research assistant to improve accuracy and reduce bias (Simons 2009). The observations focused on the conduciveness of learning venues at SACCO and administrative buildings.

For data analysis, the adult learning practices of Katureebe constituted the case and the unit of analysis. Open coding and axial coding were employed to generate categories and themes (Henning *et al*. 2004). The transcribed data were read many times and four key themes, namely learning, methods, barriers and strategies were identified as axial codes for analysis. Under learning, what Katureebe learnt such as knowledge and skills in savings, borrowing and attitudes were analysed. In the theme on methods we analysed how he learned, and this included touch–feel–recognise, and questioning. As for barriers, the hindrances to his learning and engagement with SACCOs were analysed and these included inaccessible teaching and communication approaches, unfavourable physical and psychological environment, and economic and mobility barriers. The analysis of strategies focused on the coping strategies and the support Katureebe received to address the barriers. The data analysis process was dialectical and involved assembling and disassembling of data to discover connections and relationships (Boije 2010). We as authors do not have impairments but identify with persons with disabilities’ struggles against marginalisation and discrimination. The first author has a sister with a disability and both authors have previously conducted participatory research on disability. Although Oliver (1992) proposes that persons with disability should conduct their own research, Barnes’s (2003) counter proposal is that a researcher does not necessarily need to have an impairment in order to do it.

## Ethical considerations

Ethical approval was obtained from Kyambogo University Research Grants and Publications Committee and the Uganda National Council for Science and Technology (UNCST) in Uganda. In South Africa, specifically the University of KwaZulu-Natal (UKZN), it was obtained from the Humanities and Social Science Research Ethics Committee (HSSREC). The principle of non-maleficence strongly informed our ethical considerations (Bryman 2008; Chilisa & Preece 2005; Marshall & Rossman 2011).

As Katureebe was a person with visual impairment, a category considered vulnerable, particular care was taken to ensure that he was treated with respect and in a manner that was intended not to psychologically stress or humiliate him (Bryman 2008). The checklist suggested by Mcniff, Lomax and Whitehead (1996), which includes participants’ right to withdraw, researcher trustworthiness and keeping promises, was adhered to.

### Informed consent

Informed consent was sought and obtained from the research participant orally in his local language before doing the observations or in-depth interviews, but after he was informed of his rights. The UNCST guidelines accept oral consent (UNCST 2007).

### Anonymity

Although respect for anonymity is a good ethical practice (Chilisa & Preece 2005; Rule & John 2011), Katureebe insisted we use his real name. He argued that, as he had told us the truth, there was no need to hide his identity. We agreed with him because of Nuwagaba and Rule’s (2015) argument that going against his wish would signify that his views were not being respected because of his impairment.

#### Profile of Mr. Katureebe

Mr. Katureebe was born partially blind, grew up with low vision, and became blind later in life. He lives in a peri-urban area, in Bushenyi, Western Uganda, among the tribe called Banyankore. Kinyankore culture looks down on persons with disabilities and treats them as charity cases but Katureebe is among those who show that persons with disabilities have potentials and rights. Although he lives in a largely oral society, he is adjusting to the literacy requirements of the microfinance industry.

Katureebe completed primary education, thereafter obtaining a ceramics certificate. He attributes his education to his parents’ support. He is aged 65, very intelligent and a respected leader in the community. He is vigorous for his age although sometimes he can be seen engrossed deeply in thoughts that make him unhappy. His Kinyankore name means ‘Let us see’, signifying his parents’ uncertainty about his future.

He engaged with microfinance partly because he needed additional funds for his livelihood activities, including crop farming and poultry farming (about 200 hybrid layers). Due to his old age, he stopped brick-making because it required a lot of energy. He uses a mobile phone for communication as he engages with these activities.

Katureebe, because of his visual impairment, used his hands to locate where the feeders and drinkers for his chickens were and to collect the eggs from the nesting boxes. He learnt to glide his feet on the floor as he walked instead of lifting them up, thus avoiding crushing the eggs that lay scattered on the floor outside the boxes. He sent *boda-boda* (motor cycle taxi) riders to buy and bring the bags of poultry feed to the farm. His customers came to the farm, so he did not have to take eggs to the market. He had challenges counting money but developed strategies to address them.

Katureebe, because he is a trusted member of his community, served as a treasurer of one of the Rotating Savings and Credit Association (ROSCAs) [very small microfinance groups at community level where funds are lent to members in turn] whose membership comprised persons with and those without disabilities. As his financial needs and capacity grew, he joined a SACCO and obtained microfinance loans a number of times, the highest being 1,000,000 Uganda shillings ($400). SACCOs are bigger microfinancial institutions than ROSCAs.

Katureebe desired to learn how to read and write Braille but was constrained by lack of learning opportunities and the costs involved. He has had many challenges in life. He was forced to abandon his job with a shoe company because of his impairment and his emotions are evident as he tells his story. He acknowledges that disability has made it difficult for him to realise his full potential, yet the responsibility of maintaining the family rests with him as man because he lives in a patriarchal society. The same society bestows on him control over family resources.

Katureebe faced barriers such as inaccessible teaching and communication approaches, unfavourable physical and psychological environment, and lack of finances that limited his learning and engagement with microfinance. He strove to overcome these barriers, with support from fellow persons with disabilities, SACCOs, family and community members.

## Results

Katureebe was found to have participated in two types of adult learning about microfinance – informal and non-formal. Rogers (2003) notes that informal learning occurs as a result of interaction between adults as they engage in activities in their everyday lives. Non-formal learning, on the other hand, is structured in terms of learning objectives, learning time or learning support and is intentional from the learner’s perspective.

### What Katureebe learnt and how he learnt it

Katureebe learnt a wide range of skills, knowledge and attitudes, including farming, identifying currency, getting feedback during communication, leadership roles, use of mobile phones and strategies to address barriers to participation and utilisation of microfinance services. The focus of this article is on the learning related to his engagement with microfinance.

#### How Katureebe learnt

The various ways through which Katureebe learnt depended on the interaction between his impairment, the environment and what he was learning.

**Touch–feel–recognise:** A strategy of touch–feel–recognise was used in learning how to manage money as described by Mr Katureebe:

‘I feel the sizes of the notes with my fingers after arranging them (one on top of the other) on my palm and distinguish them by size to determine their value. I know the notes are in the values of Shs 1000, 2000, 5000, 10000, 20000 and 50000 and the sizes of the notes correspond to the values of the notes.’

‘The Shs 500 coin has the shortest circumference, is thickest and has an embossed picture of the head of a bird (Authors: crested crane – a symbol on the national court of arms), the Shs 200 coin is somehow thin with a medium circumference and has an embossed picture of a fish, and the Shs 100 coin has the longest circumference, is thinnest and has an embossed picture of a cow.’

This explanation indicates a process involving touching, followed by feeling and then recognising, which Nuwagaba (2012) coined as the ‘touch–feel–recognise’ method of learning. This method served Katureebe well in identifying Ugandan currency notes, which have no embossed features. It is noteworthy that while size distinguishes the notes, thickness distinguishes the coins. He acknowledged that this method could only be used when one had all the notes and the amount involved was not large.

**Questioning:** As a learner, Katureebe used questioning as a strategy of seeking feedback while communicating with those conducting the training and fellow learners during the learning process. He mentioned that, having become blind later in life, he was aware that people often show they are following a discussion through non-verbal cues, which blind people do not pick up. His strategy was to constantly ask: *Ummmhhh? Aaaahhh? Eeee? Tukwe?* (Isn’t it?), *Onyine?* (Are you with me?). He often did not complete the words or sentences and encouraged the listener to complete them by asking: *Ummmhhh?* He then used that response as feedback to gauge whether someone was following the discussion or not.

**Practice:** Katureebe intimated that the leadership skills he exhibited as leader of their ROSCA were acquired through practising the skills acquired from the CBR training he received in 1990 saying: ‘When CBR was introduced from Bushenyi, they educated us, … we made groups and from there we who got into leadership positions started practicing leadership skills’.

The skills he mentioned included chairing meetings, making decisions regarding admission of members, communicating on behalf of their group, representing their group in leadership training and other meetings among others.

### Barriers to leaning and engagement with SACCO services

The barriers that Katureebe faced included inaccessible teaching and communication approaches, unfavourable physical and psychological learning environment, and lack of finances which limited his participation as follows:

#### Inaccessible teaching and communication approaches

Katureebe could neither see facilitators’ demonstrations during the learning process nor effectively participate in learning about microfinance services because the facilitators were using inaccessible teaching and communication approaches. This restricted his participation in learning. He noted:

‘But for someone who is blind, when your arm is up, they will point to you and say “you”, but you will not rise to contribute. They will say they meant you (pointing to you again) and again you will not know. Now you hear your neighbour saying to you and pulling your shirt: “it is like they mean you”. Now because you have first had to listen to this one [*the facilitator*] and that one [*your neighbour*], what you had wanted to say gets out of your head. Those things also stop us from going for meetings.’

Restricted participation in learning caused confusion and resulted in a loss of confidence and concentration, which is an affective barrier. Additionally, a facilitator who does not know how to communicate with adult learners with visual impairments may interpret the unintended actions of such a learner as not responding when called upon lack of seriousness or interest in learning.

Participation restrictions caused by barriers were compounded by his inability to read and write. He experienced the challenges of not being able to keep records of what was happening during learning sessions. He mentioned that he could not read any print materials that were used during teaching. This suggests that inability translates into some inadequacy in learning situations which do not cater for his needs.

Katureebe expressed grief that his inability to use Braille made him miss a lot during the learning process:

‘Writing is my problem, it is what disturbs me … The information I have is what I retain by cram work. Eeeh-something I forget or which I miss during the learning session is gone in a flash [*Nikinguruka*]. It may not be possible to ask a guide: “When they came to this part, what did they say?” This is because sometimes you find that the guide was not attentive, so the information you miss, that means you have missed it forever. ‘

Katureebe noted that barriers went beyond the teaching and learning encounter and included obstructions caused by society’s practice of queuing for food at training events. He lamented:

‘But it pains you when they say that you should all stand to join a line, not so? You smell the scent of the food, others have already started eating and they are swallowing and for you, you are swallowing saliva! Put yourself in that situation - by the time you get the food, the food will no longer be tasty because your appetite will have already been taken by others, ummmh?’

The interconnectedness of physical, affective and social elements is evident here. Society, by using a system of lining up for food, creates a physical barrier which stops him from collecting the food himself, leading to loss of confidence, which is an affective barrier, and conditions that do not allow him to stand in the line with others create a social distance between him and the others. Each of the barriers independently, as well as the combination of the barriers, affected his preparedness to learn.

## Unfavourable physical and psychological learning environment

The area where Katureebe lived was very hilly and very slippery during the rainy seasons. The buildings where microfinance training took place had accessibility and usability issues. Many had entrance steps and/or were located in steep-slanting landscapes, and inside there were steps, and cemented or tiled floors, which made use of walking sticks on them problematic. ‘I will not go there’, said Karureebe of learning events in inaccessible venues, ‘because that place is not friendly to me’.

The psychological environment was evaluated as favourable at certain times and unfavourable at other times. The psychological conditions he described as favourable included those that put him at ease with himself and with the facilitator (Gravett 2005).

Katureebe experienced a sense of inferiority and fear (affective barriers that are part of psychological barriers) which hindered or prevented his participation in learning. He added that some community members and development workers took people with impairments to be of low status, which limited their participation in learning and utilisation of SACCO services. He remarked, ‘Persons with disabilities who think that their potential and abilities are inferior, fear to mix with others and this hinders cooperation in learning sessions’. We see here the phenomenon of persons with disabilities internalising the attitudes of the able-bodied towards them, which in turn disables them as participants. This reveals that social attitudes have affective consequences.

Katureebe added that persons with disabilities were afraid of getting loans, saying:

‘We fear to get the money for a loan, and then you fail to manage the project. I want to show you an example, like me, I have a pig which I all along thought was pregnant. If I could see, I would have known that it is not pregnant and taken it where? To a male for servicing … when they [*chicken*] were attacked by a disease – coccidiosis - you are supposed to tell this disease by the sight of their droppings. Now if you don’t have an active person at home, they will be attacked by disease and die … Because I might put money in a project and I fail to manage it, and I find myself in a loss, why should I ask for a loan? For what? … You decide to die in poverty instead of being taken to prison for failure to pay back a loan. Are you following me?’

There were occasions when the physical environment influenced Katureebe’s affective state. As explained above, lining up for food in a crowded room that in addition had steps, negatively affected his mood for learning. An unfavourable physical and psychological environment was therefore a hindrance to the learning process. The absence of mobility systems or support (Scholl 1986) accentuated the environment’s hindrances. Again here we see the creation of disabling social barriers to learning, with accompanying psychological and affective dimensions, which hinder participation.

### Financial barriers

Katureebe noted that, because of limited income, he could not afford to pay membership fees of groups, transport costs to learning venues, or buy batteries for his radio all the time and so he missed SACCO-related messages. He could not afford mobility assistive devices such as white canes or a guide and all these combined negatively affected learning about and utilisation of SACCO services. He explained that, because of their impairments and unfavourable environment, persons with disabilities spent more to access services which they could not often afford. He argued, ‘As a blind person – … on top of his blindness he needs a guide. Eeh? Now life becomes double expensive’.

In addition, Katureebe was aware that lack of funds denied persons with disabilities sureties when they were applying for loans because would-be guarantors were afraid that persons with disabilities lacked the resources to pay back loans. Findings suggest that, while mobility was a barrier in its own right, it was also linked to economic barriers.

All these difficulties, however, did not deter Katureebe as his philosophy was that disability is not inability and that, through hard work, one can maintain oneself.

### Strategies to address the barriers

The strategies that Katureebe developed to overcome barriers, such as the touch–feel–recognise method, questioning and practicing, were explained in the section on how he learnt.

Other strategies, which we now turn to, included engaging in advocacy, having his daughter assist him in learning sessions and sitting close to people who knew he had visual impairment and would assist him when the need arose. He said:

‘In a learning session, when I put my hand up to contribute, some community members help attract the attention of the facilitator to give me an opportunity. They pull my shirt or pinch my ribs to alert me that the facilitator wants me to make a contribution.’

Katureebe mentioned that he relied on assistance from his daughter who listened in and observed demonstrations and posters during learning sessions, and later explained them to him. He added, ‘She helps me put a pen on the right spot which made it possible for me to sign in an appropriate place’.

Katureebe, because of his success in engaging with SACCOs, was a role model in sensitising SACCOs on how to improve access to microfinance by persons with disabilities. He was an example of how reducing discrimination against persons with disabilities can make them successful. As a result of this and efforts from disabled peoples’ organisations, SACCOs had started to provide faster services to persons with disabilities and, together with family and community members, provided personal services to Katureebe to address mobility and communication barriers. To overcome financial barriers, the government had introduced disability grants, National Agricultural Advisory Services (NAADS) and other initiatives.

It is therefore evident that Katureebe overcame some of the barriers on his own, and with others he was supported by SACCOs, family, community members and the government. He also helped other people with disabilities to overcome barriers. The principle of interdependence emerges from these strategies as important in overcoming barriers.

On the whole, the data do not seem to show any difference in learning approach, barriers to learning or strategies to overcome the barriers whether Katureebe was learning informally or non-formally.

## Discussion

Our discussion begins with the issue of a case study and generalisability, particularly regarding a case study of a single subject. It then follows three lines of argument: barriers to learning and microfinance existed for a person with visual disability in Uganda; the social model of disability sheds light on such barriers; and the barriers can be overcome through active engagement and interdependence.

A ‘limitation’ of case study findings that is frequently cited is their lack of generalisability. However, case study experts have countered that statistical generalisation is not the point of case study. As Yin (2003:32) argues, a case study can generate ‘analytical generalisations’ in which generalisations are made to theoretical propositions rather than populations. In this paper, the findings regarding the social factors that constrained Katureebe’s learning confirm the theoretical template of the social model of disability as a way of understanding the experiences of adult learners with disabilities regarding microfinance, as we show below. Besides testing existing theory, a case study, even the rich and detailed account of a single subject, as in this paper, can generate new insights into a phenomenon (Rule & John 2011; Simons 2009). For example, Katureebe’s experiences point to the importance of psychological responses to constraining social conditions as a key factor that impacts on learning of the individual person with a disability. Applying this finding more widely could happen through a process of what Thomas (2010:577) calls ‘abduction’ and ‘phronesis’. Here, the focus is on generating practical wisdom from a case study which readers can then apply to related cases and make discerning judgements about appropriateness. This paper sheds light on the experiences of a single participant with a visual impairment regarding learning and microfinance. As such, the findings yield insights, through discerning application, into the nexus of disability, microfinance and adult learning, without succumbing to the claim that Katureebe is a representative of all adults with disabilities involved in microfinance or that his experiences can be generalised to theirs.

Results show that Katureebe learnt to engage with livelihood, microfinance and other activities through various methods such as touch–feel–recognise, questioning and practice. However, despite his motivation, capacities and a wealth of experience, Katureebe faced a range of barriers such as inaccessible teaching and communication approaches, unfavourable physical and psychological environment, and limited finances. These barriers were interconnected and impacted on each other as they influenced Katureebe’s mobility and learning activities.

The first category of barriers that hindered his learning was identified as negative attitudes of community members who regarded persons with disabilities as inferior. This is consistent with Naami’s (2014) findings in Ghana regarding society’s negative attitudes towards persons with disabilities, doubts about their capacities and discrimination against them. Stone (1997) argues that some people view persons with disabilities as people who need to be pitied and yet they do not like it and prefer to be viewed as any other person. When they are pitied, it affects their motivation and consequently, their learning potential may be compromised. WHO (2010b) identifies negative attitudes of families and communities as some of the most damaging barriers. It is likely that such negative attitudes may create negative feelings that may inhibit learning (Zembylas 2008).

The second category was the unfavourable environment in which he learnt and engaged with microfinance. These included steep landscapes and inaccessible buildings, the kinds of barriers that Stone (1997) identified as natural and artificial.

The third category was financial barriers. Katureebe raised collateral with great difficulty and could not access messages about microfinance and other opportunities because, often, he could not afford dry cells for his radio. Inaccessible information about learning and microfinance opportunities constrained his participation in SACCOs thus compounding his financial barriers. Indeed economic factors limit many persons with disabilities’ participation in educational and other development activities (Lwanga-Ntale 2003; MoFPED 2008; Naami 2015; Stone 1997).

These three categories of barriers are clearly a societal creation as explained by the social model. Viewing the barriers through the social model of disability, Oliver (1996) reveals that persons with disabilities were discriminated against by able-bodied society (Clark 2006; Naami 2014). The study confirms that, despite good legislation, barriers still existed in practice in Uganda. Similar results had been found earlier in Uganda (Nuwagaba *et al*. 2012) and in Ghana (Naami 2014). The study confirms Oliver’s (1996) argument that economic, cultural, attitudinal, physical and social barriers disable persons with impairments.

The fact that Katureebe’s learning about SACCO services was possible with support from his family, SACCOs and community members is testimony that society which erects barriers is capable of removing them. This could be performed, for example, through supporting the person with impairment and providing a disability-friendly learning environment, thus enabling persons with disabilities access to services and empowering them. This strengthens the social model’s explanation of disability as a social phenomenon (Oliver 1996) and the holistic approach to CBR as proposed by the Convention on the Rights of people with disabilities of 2006. However, Watson (2004) criticises the social model for presenting an incomplete picture of disability. For example, it cannot explain Katureebe’s personal limitations and impairment and how they affected his learning. He notes that socialisation of disability shifts the focus to commonalities, thus underplaying the complexity and diversity of each person’s lived experiences (Watson 2004:101). Also, society had accepted Katureebe as a role model because he had successfully learned and engaged with SACCOs and he shared his testimony during training programmes. As National Union of People with Disabilities (NUDIPU) (nd) and AMFIU (2010) suggest, supporting persons with disabilities to share their experiences during learning, and in other development activities, can help improve their participation.

The fourth category, Katureebe’s negative attitudes, indicates an interplay between the social and the personal. Social barriers may have psychological consequences, including loss of confidence and withdrawal from activities. Katureebe’s negative attitude towards himself and feeling of inferiority sometimes made him exclude himself from community activities including learning. A study in Ghana revealed that some barriers, such as low levels of self-confidence, negative reaction to societal attitudes and ignorance about their own potential, were personal rather than societal (Naami 2014). Here, we argue that the social and the personal are closely related. This is because the societal barriers as well as Katureebe’s personal barriers influenced each other and affected his learning. The societal and personal factors coupled with an unfavourable learning context further constrained his learning. Fear as a result of vulnerability has been identified elsewhere as an inherent barrier in the context of disability and HIV & AIDS (Sweeney 2004). A combination of negative attitudes, inferiority and fear, within a sometimes excluding and discriminating social context, contributed to Katureebe’s emotional disposition. Zembylas (2008) established that emotional experiences regarding injustices can sometimes inhibit learning (Zembylas 2008) and this can also result in the internalisation of oppression (Freire 1972).

Viewing Katureebe inherent barriers and those erected by society through the social model (Oliver 1996) and critical theory (Henning *et al*. 2004) indicates that these barriers combined to marginalise, oppress and discriminate against Katureebe as an adult learner. In Katureebe’s case, his positive psychological disposition motivated him to learn. In this process some of the societal barriers were removed by the society that had erected them (Clark 2006) thus further enhancing his participation in learning. The study also reveals that, despite the favourable disability policies, the reality shows that persons with disabilities are still faced with barriers.

## Limitations of the study

Although we had planned to observe Katureebe’s involvement in non-formal learning sessions, that was not possible as none occurred during the period of data collection. The data regarding learning processes may therefore have inadequacies as they are based on opinions about the process, although the physical layout and accessibility of training venues were observed. There could be differences between Katureebe’s views on the processes and the actual learning processes. In addition, one participant and his experiences cannot be generalised to represent all persons with disabilities’ experiences of microfinance in Uganda. Nevertheless, his story yields insights into some of the ways in which disability and adult learning interface with microfinance in Uganda, and as Simons (2009:9) notes, the case study helps ‘understand the case itself rather than generalise to a whole population’, as discussed above.

## Conclusions

Katureebe, as a person with visual disability, successfully engaged with learning and microfinance. However, he faced barriers to learning and engagement in livelihood and SACCO activities. The barriers were actually experienced in a combined manner but were categorised to facilitate analysis. The barriers were theorised using the social model of disability. Katureebe developed coping strategies through which he changed the conception of barriers from permanent obstacles that obstructed persons with disabilities to hurdles to be overcome. He helped others and also received help from fellow persons with and those without disabilities, and this interdependence – a key feature of the social model of disability (Oliver 1996) – helped reduce the barriers. Interdependence, unlike dependence, empowers, as each person has something to offer and to gain. Also, unlike the ‘independence’ of autonomous individuals, it shows the connectivity and relationality of persons with disabilities in an African context. This interdependence has the potential to enhance persons with disabilities’ learning and engagement with microfinance. For Katureebe, therefore, many of the barriers he faced were surmountable. Although some of the barriers could be removed, the impairments remain, meaning he still remains with some challenges such as not being able to see during learning and engagement with SACCOs. Katureebe’s case provides evidence to confirm that the social model of disability can be extended to encompass the psychological consequences of social barriers through a more interactional understanding of biological, psychological and social factors (Rule & Modipa 2012; Schneider 2006).

### Recommendations

Although our case was exploratory and based on a single case of one participant, findings can be used to suggest that adult education programmes for persons with disabilities should aim at addressing the unique learning challenges of persons with visual and other disabilities. This is because the challenges are not homogenous to all categories of disabilities but relate to disability type. Also, the persons with disabilities, as well as the communities in which they live and work, and their agencies that provide services, should be sensitised about disability. Each side has a role in addressing the barriers faced by persons with visual and other disabilities.
